# Novel Surgical Interventions for the Treatment of Obesity

**DOI:** 10.3390/jcm13175279

**Published:** 2024-09-05

**Authors:** Gerardo Perrotta, Sara Bocchinfuso, Noura Jawhar, Aryan Gajjar, Richard S. Betancourt, Ray Portela, Wissam Ghusn, Omar M. Ghanem

**Affiliations:** 1 Department of Surgery, Mayo Clinic, Rochester, MN 55095, USA; dr.gerardo.perrotta@gmail.com (G.P.); bocchinfuso.sara@mayo.edu (S.B.); jawhar.noura@mayo.edu (N.J.); aryangajjar0722@gmail.com (A.G.); rayportela10@gmail.com (R.P.); 2 Mayo Clinic Alix School of Medicine, Mayo Clinic, Rochester, MN 55095, USA; betancourt.kylerichard@mayo.edu; 3 Internal Medicine Department, Boston Medical Center, Boston, MA 02118, USA; ghusn.wissam@mayo.edu

**Keywords:** obesity, bariatric surgery, metabolic surgery

## Abstract

Metabolic and bariatric surgery is widely recognized as the most effective and durable treatment for the disease of obesity and its associated comorbidities. In recent years, the field has seen significant advancements, introducing numerous innovative surgical options. This review aims to comprehensively examine these emerging surgical techniques, which have recently received endorsement from the American Society for Metabolic and Bariatric Surgery (ASMBS). Additionally, we will explore new technologies and methodologies supported by the latest scientific evidence. Our analysis will include a critical evaluation of the efficacy, safety, and long-term outcomes of these novel approaches, providing a detailed update on the current state of metabolic and bariatric surgery, highlighting key developments and their potential implications for clinical practice.

## 1. Introduction

Obesity is a chronic and complex disease with a dramatically increasing trend, with one in two adults in the United States projected to have obesity in 2030 [1]. It is driven by multiple factors, including genetics, metabolic processes, and lifestyle behaviors, and remains a crucial modifiable risk factor for numerous serious health conditions, such as cardiovascular disease, diabetes, and cancer [2].

Obesity management has evolved significantly over the decades, influenced by advancing medical knowledge and technological innovation [3]. As such, the management strategies for obesity have diversified to include lifestyle interventions, pharmacological treatments, and surgical interventions [4], each playing a pivotal role in comprehensive obesity care [5]. The pharmacological approach to obesity management has seen substantial advancements, with several medications approved for long-term use. These medications typically function by suppressing appetite, increasing satiety, or slowing gastric emptying [6].

Recent advancements in bariatric procedures include various endoscopic techniques that offer less invasive alternatives and other bariatric surgeries that have been recently introduced into the field. Among the endoscopic methods, endoscopic sleeve gastroplasty is one of the most popular and utilizes internal sutures to reduce the stomach volume, enhancing weight loss [7,8].

Metabolic and bariatric surgery has revolutionized the landscape of obesity management, particularly for patients with severe obesity. The historical journey of these surgical procedures began with more invasive techniques, which have gradually given way to less invasive, laparoscopic methods that offer reduced recovery times and complications [9,10]. The two most commonly performed bariatric surgeries today are sleeve gastrectomy (SG) and Roux-en-Y gastric bypass (RYGB) [11]. Both procedures not only restrict food intake, but also induce hormonal changes that assist in weight loss [12].

In this study, we aim to elaborate on some of the recent and less commonly performed bariatric surgical interventions (Table 1). The single-anastomosis duodeno–ileostomy with sleeve (SADI-S) and the one-anastomosis gastric bypass (OAGB) are notable for their simplified approaches which potentially reduce surgical risks and improve metabolic outcomes. While these procedures have been reported upon for some time, they have only been endorsed recently by the American Society for Metabolic and Bariatric Surgery (ASMBS). SADI-S combines aspects of a sleeve gastrectomy with a duodenal switch (loop configuration), limiting both intake and absorption, while OAGB streamlines the traditional Roux-en-Y gastric bypass procedure to a single anastomosis, aiming to reduce surgical time and bowel obstruction rates [13]. Additionally, the innovative magnet system for duodeno–ileostomy (MSDI) introduces a magnetic device to facilitate the connection between bowel parts, aiming to reduce surgical complications and enhance precision [14]. Sleeve gastrectomy with transit bipartition (SG-TB) and single-anastomosis sleeve ileal (SASI) bypass are emerging as options that modify nutrient pathways and potentially enhance hormonal responses to treat obesity and associated metabolic disorders more effectively [15]. These procedures reflect a growing trend towards employing advanced technology and surgical techniques to potentially achieve better patient outcomes in the treatment of obesity.

## 2. Methods

An extensive literature review was performed by the authors of this manuscript in three phases: identifying relevant research questions and bariatric surgery techniques, performing the literature review, and reporting the most relevant findings. The different tasks were coordinated by all the involved authors. Using PubMed, relevant articles were chosen from inception to 2024 according to our keywords: “obesity”, “bariatric surgery”, and “metabolic surgery”. Only the most recent techniques were included, starting from those recently endorsed by the ASMBS. The selection of surgical techniques and articles was approved by the corresponding author, who provided his expert opinion to synthesize the information and edit the manuscript draft.

## 3. Single-Anastomosis Duodeno–Ileostomy with Sleeve (SADI-S)

One of the latest procedures to be endorsed by the American Society for Metabolic and Bariatric Surgery (ASMBS) is the single-anastomosis duodenal–ileal bypass with sleeve (SADI-S) [16]. While the technique was initially described in 2007 [17], its use in the United States remained unbroached until at least 2020. The Metabolic and Bariatric Surgery Accreditation and Quality Improvement Program (MBSAQIP) database reported 488 cases in 2020, and a subsequent rise in popularity to 1025 cases in 2021 and 1567 in 2022. Examining the increase in SADI-S procedures relative to the general increase in bariatric surgery during this period; this percentage has also risen from 0.24% of all procedures in 2020, to 0.39% in 2021 and 0.56% in 2022 [18].

### 3.1. Patient Selection

SADI-S is effective for long-term weight loss and remission of type 2 diabetes mellitus (T2DM), while carrying a greater risk for gastrointestinal reflux disease (GERD) and bowel complications compared to RYGB. These characteristics align with a study that compared preoperative patient characteristics between those undergoing SADI-S and RYGB and found those undergoing SADI-S having higher body mass index (BMI) and incidence of hypertension, but a lower incidence of GERD [19]. Another advantage is the utility of SADI-S as a revisional procedure for patients with previous SG. One study at an academic tertiary referral center compared the efficacy of revisional SADI-S and OAGB in weight loss and comorbidity resolution. At 5 years postoperatively, SADI-S was found to have statistically significant greater change in BMI and total weight loss (TWL) with similarly beneficial results for excess weight loss (EWL). SADI-S also showed higher rates of T2DM resolution than OAGB (75% in the SADI-S group vs. 50% in the OAGB group) [20].

### 3.2. Technique

**Figure 1:**
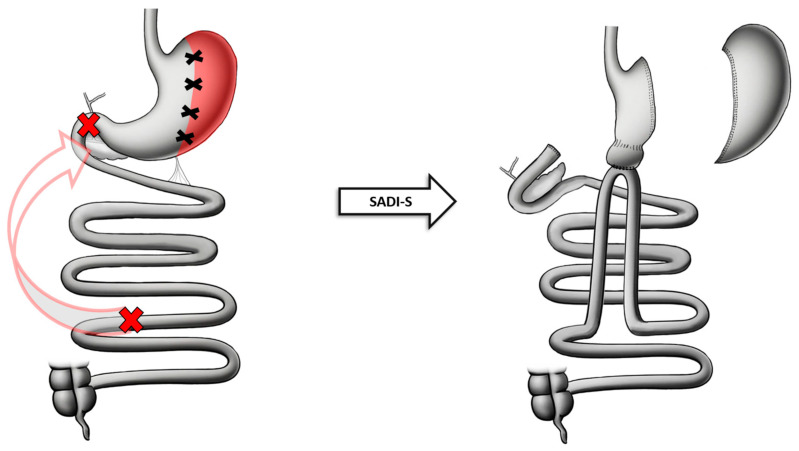
Anatomical configuration of SADI-S.

The initial step in SADI-S is the opening of the gastrocolic ligament and the dissection of the greater curvature vasculature of the stomach from the body and to fundus proximally and beyond the pylorus distally. An SG is then performed according to standard technique, but with a larger diameter calibration tube (50–54 Fr). Following identification of the ileo-cecal valve, a distance of 300 cm proximally is measured, marking the site where the anastomosis will take place. After transecting the proximal duodenum just distal to the pyloric sphincter (typically 2–3 cm), the marked ileal loop is anastomosed to the proximal sectioned duodenum (Figure 1) [21]. Advantages of this technique are less tension in the loop limb, the presence of a single anastomosis, reduction in ulceration risk, and a decreased post-prandial surge by virtue of retaining the pyloric sphincter [22,23].

### 3.3. Safety and Efficacy

Compared to RYGB and OAGB, SADI-S was found to have higher TWL at 1, 2, 3, and 5 years [24]. Similar results were shown in a multicenter study of patients with very high BMI (BMI ≥ 50 kg/m^2^), with SADI-S demonstrating higher TWL than RYGB at 6, 12, 24, and 60 months postoperatively and higher resolution rates of most obesity-associated comorbidities (hypertension, T2DM, and dyslipidemia), proving to be an excellent option in this cohort [25]. When compared to biliopancreatic diversion with duodenal switch (BPD-DS), SADI-S was shown to have similar comorbidity resolution rates and lower complication rates, despite a slightly lower excess BMI loss [26]. Other studies have found similar results regarding the efficacy of SADI-S for weight loss and comorbidity resolution [27,28].

While initial reports indicated higher complication rates with SADI-S when compared to RYGB (but 30-day outcomes comparable to BPD-DS) [29], more recent studies revealed a similar or better safety profile, with SADI-S having lower rates of marginal ulceration, anastomotic leak, and internal herniation than RYGB [25], suggesting that earlier reports were due to the learning curve of this relatively new procedure (vs. the superior and safer results in the more experienced tertiary centers). The lower rates of marginal ulceration with SADI-S can be explained by the protective role on the ileal mucosa of the alkaline fluid secreted by Brunner’s glands [22], while the lower incidence of internal hernia, when compared to RYGB, is likely due to the presence of a single anastomosis and the avoidance of two mesenteric openings (only one in SADI-S). Although meta-analysis has shown it to be a rare occurrence, concerns regarding long-term risks of bile reflux remain, and more studies with longer follow-up are needed to better evaluate the risk [30].

### 3.4. Conclusions

SADI-S is a safe and effective procedure that has shown impressive results with sustained weight loss and resolution of comorbidities, with decreased complication rates. It is therefore expected that it will continue to gain popularity in the United States and in other countries where it is currently underutilized.

## 4. One-Anastomosis Gastric Bypass (OAGB)

One-anastomosis gastric bypass, also known as omega loop gastric bypass and mini gastric bypass, was first introduced in 1997 as an alternative technique in bariatric surgery that is both safe and effective while reducing operative times [31]. This technique was improved upon by Carbajo and Caballero to reduce bile reflux [32]. Since then, the procedure has been gaining traction worldwide, especially in Europe and Pacific Asia. According to the International Federation for the Surgery of Obesity and Metabolic Disorders (IFSO), the standard nomenclature for this procedure is one-anastomosis gastric bypass (OAGB) [33].

### 4.1. Patient Selection

Patient selection is made according to criteria by the National Institutes of Health Development Panel (BMI > 40 kg/m^2^ or BMI > 35 kg/m^2^ with severe related comorbidity). Patients generally present with a higher preoperative BMI and comorbidity prevalence when compared to those receiving RYGB, as OAGB is a hypoabsorptive procedure [34]. Preoperatively, all patients should undergo a thorough assessment involving multidisciplinary psychological, nutritional, and medical evaluations. As with SADI-S, patients with severe reflux might not be the best candidates for OAGB [35].

### 4.2. Technique

**Figure 2:**
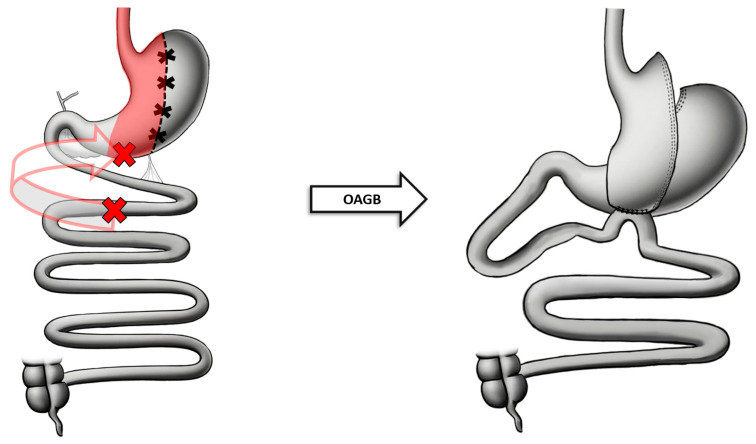
Anatomical configuration of OAGB.

The OAGB procedure is performed laparoscopically or in a minimally invasive fashion and consists of two phases: first, a restrictive gastric pouch is created; secondly, a malabsorptive antecolic gastrojejunostomy anastomosis is formed [32]. In the first phase, the creation of the gastric pouch is initiated by entering the lesser sac 2–3 cm below the crow’s foot (the terminal posterior gastric branches of the posterior vagal trunk). The stomach is divided with a stapler at a 90° angle to the lesser curvature at this point. Staples are used to divide the stomach parallel to a 36 Fr calibration tube moving proximally toward the angle of His, effectively excluding the fundus. This creates a narrow longitudinal (tubular) pouch that should be approximately 13–18 cm long. In the second phase, the jejunal bypass is created. The ligament of Treitz is identified and the length of the small bowel is measured from this point to ensure that the biliopancreatic (BP) limb is 150–200 cm long. The jejunum at this location is brought cephalad to create an antecolic end-to-side anastomosis with the gastric pouch. The anastomosis is tested using an air leak test prior to closure (Figure 2) [36].

### 4.3. Safety and Efficacy

IFSO released updated OAGB recommendations in 2021 [33] based on positive outcomes observed in prior studies with regards to operative times, complication rates, weight loss, and remission of obesity-related comorbidities. OAGB as a primary procedure appears to be at least equivalent to other bariatric procedures, including RYGB. Reported mean operative times range from 37 to 130 min [31,32,34,36,37,38,39,40,41], with the shortest reported time being 19 min [31]. In comparison, laparoscopic RYGB mean operative time was reported as 113 min and is, on average, longer in duration than OAGB [34]. Mean hospital stay is reported to range from 1 to 5 days [37,38,42,43], which is comparable to RYGB [37,40,41].

A randomized multicenter open-label trial comparing outcomes of OAGB vs. RYGB demonstrated noninferiority of OAGB when comparing mean percentage excess BMI lost at 2 years [35] (−87.9% in the OAGB group vs. -85.8% in the RYGB group). Studies have demonstrated that OAGB is equal to RYGB in terms of EWL [37,38,39,40,41,44,45,46,47], at least up to 5 years postoperatively [40,41,42]. This is true when compared to SG as well [48]. EWL has been reported as high as 89% at 1 year [43], 88% at 2 years, with 70% after 12 years [36], demonstrating long-term maintenance of weight loss. In the same study with a 12-year follow-up, mean BMI decreased from 46 kg/m^2^ preoperatively to 28.5 kg/m^2^ at 6 years and 29.9 kg/m^2^ at 12 years [36]. Substantial BMI reduction and EWL were also associated with improvement in overall Gastrointestinal Quality of Life Index score [49].

Studies have demonstrated improvement in major obesity-associated medical illnesses following OAGB that is either equal to or more beneficial than RYGB [34,35,36,37,39,40,41,42,44,45,46,49] and more beneficial than SG [48]. These comorbidities include T2DM, hypertension, dyslipidemia, liver steatosis, and sleep apnea. Lower HbA1c values have been reported following OAGB compared to SG in patients with diabetes attributed to higher incretin effect [50].

It has been suggested that there is no significant difference in adverse events when OAGB is compared with RYGB [45] and that OAGB may even be associated with reduced postoperative pain [37]. Early minor complications have been reported in approximately 2–4% of patients [38,43], while early major complications have been reported in 2–3% [42,43]. Higher complication rates are seen in patients who have had prior bariatric surgery [43]. The most common early complications include bleeding (occurring in < 3% of patients [42]), anastomotic or gastric pouch leak (occurring in 0.1–1.9% of patients [42], which is a similar incidence to RYGB [34,40,41]), and early small bowel obstruction. Cardiopulmonary complications, such as pneumonia, myocardial infarction, or pulmonary embolism, occur very rarely [51].

Late complications include marginal ulceration, reported to occur between 0.6–4% of patients [42], which is comparable to RYGB [52]. The incidence of dumping syndrome is reported to be similar to [41] or less than [34] RYGB. The incidence of internal hernia and bowel obstruction appears to be smaller than following RYGB [34,40,41], likely due to the fact that OAGB does not create two mesenteric defects that predispose to this complication (only one defect in OAGB). A study analyzing GERD following OAGB demonstrated that OAGB did not increase intragastric pressure or gastroesophageal pressure gradient, and therefore did not increase the incidence of GERD [53], although there is a reported higher incidence of bile reflux following OAGB when compared to SG and RYGB [34,48]. There are no long-term data to support an association between OAGB and esophageal and gastric cancers; however, there is a feared empiric risk due to prolonged exposure of the gastroesophageal mucosa to bile, which has been shown to correlate with malignancy over time [42]. In an updated position statement, IFSO recommends regular endoscopic screening for upper gastrointestinal tract cancers in patients who have undergone OAGB [33].

Nutritional deficiencies are a main concern after OAGB and are reported more frequently following OAGB compared to RYGB, and include malnutrition, hypoalbuminemia, iron-deficiency anemia, hypocalcemia, and fat-soluble vitamin deficiency [40,41,44,54]. Iron-deficiency anemia has been reported in up to 10% of patients following OAGB [42]. Malabsorption occurs in 1.1% of patients [42]. BP limb length appears to be an important determining factor in the magnitude of nutritional deficiencies following OAGB, with longer BP limbs associated with a greater magnitude of weight loss and resolution of obesity-related complications, at the expense of nutrition-related complications, such as anemia [38]. IFSO recommends annual nutritional review, to ensure adequate nutritional supplementation [33]. Fortunately, most patients will respond to supplementation alone, without the need for additional surgical procedures [42].

### 4.4. Conclusion

OAGB is a promising alternative to RYGB for bariatric patients. For those who undergo this procedure, we agree with the IFSO guidelines stating that patients should be encouraged to remain in long-term multidisciplinary care to ensure adequate nutrition and to allow more long-term follow-up studies [33].

## 5. Sleeve Gastrectomy with Transit Bipartition (SG-TB)

Due to the evolving understanding of digestive physiology and the associated interaction with neuroendocrine signals, the technique of bipartition was developed [55]. Santoro et al. first proposed the sleeve gastrectomy-transit bipartition (SG-TB) in 2006 as an alternative technique for malabsorptive bariatric procedures and showed a favorable short-term safety and efficacy profile of the procedure with positive weight loss outcomes and obesity-related medical condition resolution and improvement [55]. The procedure was initially described as a solution to the malabsorptive nature of the biliopancreatic diversion (BPD). Santoro et al. conducted the procedure by performing a standard SG, followed by a gastric–ileal anastomosis and concluding with an ileal–ileal anastomosis [56]. This technique allows for an early diversion to the ileum while maintaining access to the duodenum, which permits quick digestion, amplifies the nutrition signal of the distal bowel, and diminishes the exposure of nutrients to the proximal bowel [57]. The lack of small bowel exclusion, especially duodenum and jejunum, prevents intense mechanical restriction and decreases the possibility of malabsorption and nutritional complications while also preserving integral neuroendocrine stimulation [58].

### 5.1. Technique

**Figure 3:**
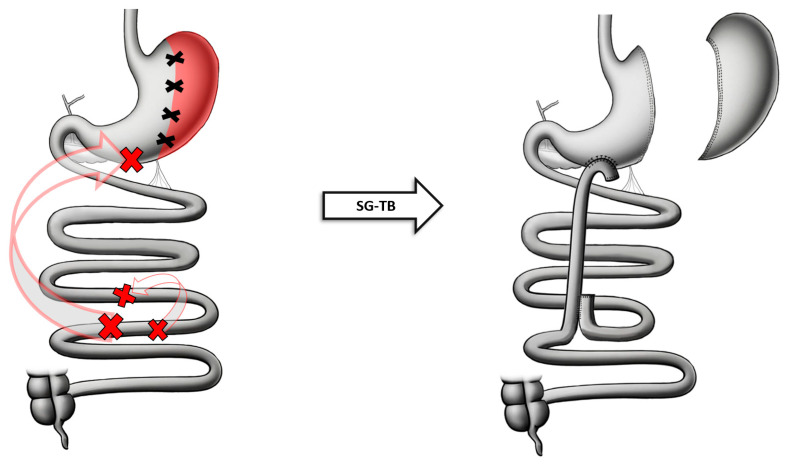
Anatomical configuration of SG-TB.

An SG is performed by freeing the greater curvature from 2 cm proximal to the pylorus up to the angle of His. The linear stapler starts at the greater curvature at 4 to 5 cm from the pylorus up to 0.5 cm from the angle of His. A 36 Fr bougie is passed through the stomach to ensure a 3 cm wide internal lumen of the remnant gastric tube and the staple line is buried with a seromuscular running suture. Once the SG is performed, the ileum is identified and marked 260 cm and 80 cm from the ileocecal valve, respectively. The distal ileum (the point at 260 cm) is anastomosed with the gastric antrum and a latero–lateral single-layer gastroileal anastomosis in an antecolic position is created with a diameter of 3 cm. A running suture closes the residual defect. In the following step, the ileal–ileal anastomosis is performed by anastomosing the small bowel cranial to the gastro–ileal anastomosis to the ileum at 80 cm proximal to ileocecal valve in a lateral–lateral fashion. The segment between the two anastomoses is then interrupted with stapling and cutting. All mesenteric defects are then closed to avoid the occurrence of internal hernias [56] (Figure 3). More recently, a loop configuration format of the same procedure was developed.

### 5.2. Patient Selection

This procedure is typically reserved for adult patients aged between 18 and 65 years with primary morbid obesity with BMI ≥ 50 kg/m^2^ with or without obesity-related medical conditions such as T2DM, hypertension, dyslipidemia, and obstructive sleep apnea (OSA). Due to the novelty of this procedure, lack of duodenal exclusion, and known long-term complications of commonly performed bariatric procedures, patients with poorly controlled T2DM are ideal candidates, especially when SG is not advised.

### 5.3. Safety and Efficacy

Santoro et al. reported a successful 5-year safety and efficacy profile of SG-TB by revealing a maintained 74% excess body mass index loss (EBMIL) in 1020 patients [56]. Topart et al. showed that while BPD-DS was superior in terms of maximum weight loss at 1 year postoperatively with an EBMIL of 83.7% vs. 78.6% and a TWL of 54.7% vs. 41.3%, SG-TB resulted in fewer complications during the postoperative period [59]. Another study by Topart et al. reported that when SG-TB was compared to RYGB performed in patients experiencing severe obesity, SG-TB led to significantly sustained weight loss with EBMIL of 85.3% vs. 73.9% and TWL of 44.8% vs. 38.4%. SG-TB patients also developed less severe surgical complications and had a lower reoperation rate within the first 2 postoperative years [60]. Al et al. observed similar trends in EBMIL, with an EBMIL of 87.7% and TWL of 20.2% in 335 patients within the first 2 years after surgery [61].

Regarding the remission of obesity-related medical conditions, Santoro et al. reported significant remission rates of 86% for T2DM, 72% for hypertension, 85% for dyslipidemia, and 91% for OSA [56]. Topart et al. revealed an 80% remission rate for T2DM, 77% rate for hypertension, and 88% rate for OSA in 71 patients over 2 years. Regarding improvement of diabetes, there were no significant differences between BPD-DS, SG-TB, and RYGB [59,60]. Similarly, Al et al. found an overall remission rate of 79.2% for T2DM [61].

The safety profile of SG-TB was examined by Santoro et al. with a follow-up of up to 5 years, revealing an early (30-day) complication rate of 6%, 1.9% reoperation rate, and 0.2% mortality rate. Significant early postoperative complications included fistula (0.9%), intestinal subocclusion (0.8%), prolonged ileus (0.7%), incisional wound dehiscence (0.4%), and intraperitoneal infection (0.3%). Late complications, occurring more than 30 days following the surgery, included intestinal obstruction due to adhesions or internal hernias (2.4%) and incisional hernia (3.1%). Most patients experiencing some form of intestinal obstruction did not present with typical symptoms or signs of obstruction due to the presence of the TB. The most common complication reported following SG-TB was diarrhea. The incidence of gastro–ileal ulcers or stenosis at the gastro–ileal anastomosis was rare [56]. Due to the lack of duodenal and jejunal exclusion, protein malnutrition, malabsorption, chronic hypoalbuminemia, and chronic anemia did not occur, and SG-TB patients presented with excellent and stable nutritional status during the postoperative period [56]. Topart et al. did not report a statistically significant difference in the overall early complication rate between SG-TB and RYGB (4.2% vs. 5.6%). However, the study did conclude a greater late complication rate within the first 2 postoperative years following RYGB [60]. Al et al. observed an overall complication rate of 10.2% after SG-TB and noticeably reported a 0% mortality rate [61].

## 6. Single-Anastomosis Sleeve–Ileal Bypass (SASI)

The single-anastomosis sleeve ileal bypass (SASI) is a simplified alternative to the SG-TB procedure first reported in 2016 by Mahdy et al. [58]. Similarly to other single-anastomosis procedures, the SASI can cause possible biliary reflux. However, the procedure itself maintains the normal pathway of food.

### 6.1. Technique

**Figure 4:**
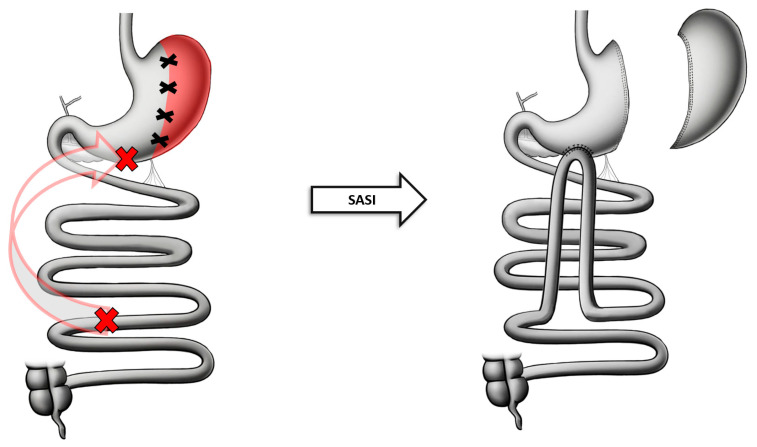
Anatomical configuration of SASI.

Once completing the SG and creating the gastric pouch as mentioned above, a single-antecolic side-to-side gastroileal anastomosis is created. The ileocecal valve is identified, and 250–300 cm of small bowel is measured proximally. This ileal loop is brought over to the antrum without dividing the mesentery and the anastomosis is created between the anterior wall of the gastric antrum, 3–5 cm away from the pylorus, and the ileal loop. The created anastomosis would be 3 cm in diameter and the anterior wall of the gastroenterostomy is closed with running sutures. All mesenteric defects are then closed to avoid the occurrence of internal hernias [58,62] (Figure 4).

### 6.2. Safety and Efficacy

Mahdy et al. first revealed the efficacy of SASI in 2016 and showed that, within the first postoperative year, the EWL achieved was 90% among 50 patients [58]. Another study conducted by Mahdy et al. reported sustained reduction in BMI and that the TWL and EWL were greater following SASI than after OAGB and SG [63,64]. When comparing SG and SASI, Emile et al. found that at 6 months postoperatively, patients who underwent SG and SASI had similar EWL [65]. However, at 1 year following surgery, EWL within the SASI patient group (72.6%) was significantly higher than the EWL observed in patients who underwent SG (60.4%) [65]. A more recent study of 116 patients in 2022 by Hosseini et al. reported that SASI leads to sustained weight loss with an EWL of 80.6% and TWL of 31.81% 3 years following surgery [66].

SASI was also successful in achieving remission and sustained improvement of obesity-related medical conditions. According to Mahdy et al., SASI resulted in an 83.9% complete resolution rate of T2DM in 551 patients, with 65% of patients experiencing improvement in dyslipidemia, 36.1% in hypertension, and 57.8% in OSA [64]. The study also found that SASI led to improvement in GERD, with 92.1% of patients reporting such improvement 1 year after the surgery [64]. Mahdy et al. reported that most patients did not show any signs of diabetes at 3 months postoperatively, with a 100% improvement rate [58]. Upon comparison, SASI was superior to OAGB and SG regarding improvement in T2DM, with an improvement rate of 97.7% vs. 86.7% vs. 71.4% [63]. Khalaf et al. revealed a 97.9% remission rate for T2DM, 70.4% for hypertension, and 100% for OSA in 322 patients over 2 years [67]. The study also found an 80.7% remission rate for GERD. In comparison to SG, Emile et al. reported a 95.8% remission rate for T2DM following SASI compared to a 70% rate following SG [65]. In terms of GERD improvement, Emile et al. found that 85.7% of patients with preoperative GERD reported improvement, while only 18.2% of SG patients experienced improvement [65].

The systematic review by Emile et al. reported an overall complication rate of 12.3% (116/941), 10.4% of which were minor complications and 1.9% were classified as major complications. The complications included bile reflux/bilious vomiting (3.3%), marginal ulcer (0.85%), bleeding (0.74%), obstruction (0.43%), dumping syndrome (0.43%), leak (0.32%), GERD (0.2%), and incisional hernia (0.11%) [62]. The study concluded that a longer common limb of 300–350 cm rather than 250 cm was associated with significantly fewer complications (3.9% vs. 14.2%) due to less malabsorption, and a lower EWL at 6 months, while a larger anastomosis size of 4 cm was correlated with fewer complications (2.6% vs. 14.1% seen in 3 cm) and greater weight loss at 6 and 12 months [62]. The incidence of nutritional deficiencies was found to be low, with an occurrence rate of 1.3% for hypoalbuminemia and 0.2% for hypocalcemia. The mean follow-up of the studies evaluated within the systematic review was 12 months [62]. Mahdy et al. observed a 30-day readmission rate of 0.72% with an overall complication rate of 10% [64]. The nutritional profile of patients following SASI and RYGB with a long BP limb was also compared by Mahdy et al., and showed that 1 year after RYGB, the levels of iron and serum albumin were substantially decreased, which was not seen in the SASI patient group [57]. Emile et al. suggested that a single anastomosis and a channel length of 300 cm might reduce the prevalence of protein malnutrition and malabsorption, which have been frequently linked to the SASI [68]. Future research should focus on standardizing the SASI approach to establish an effective solution to addressing nutritional deficiencies.

## 7. Single-Anastomosis Sleeve-Jejunal Bypass (SASJ)

The single-anastomosis sleeve jejunal bypass (SASJ), an alternative to the SASI introduced in 2019, is performed using a shorter BP limb length compared to SASI to prevent long-term nutritional complications.

### 7.1. Technique

**Figure 5:**
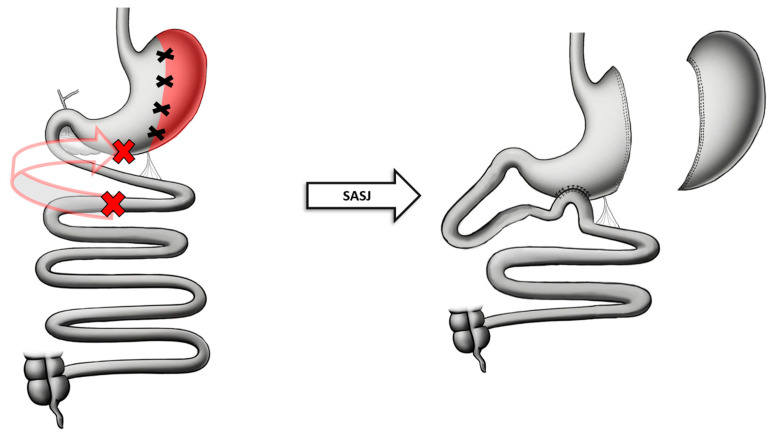
Anatomical configuration of SASJ.

Following the creation of the SG, the duodenojejunal junction is identified and a point 200 cm from the ligament of Treitz is marked. This intestinal loop is brought up to the sleeve and a side-to-side anastomosis is performed 4–6 cm away from the pylorus. The remaining defect is closed with a two-layer running suture. All mesenteric defects are then closed to avoid the occurrence of internal hernias [69] (Figure 5).

### 7.2. Safety and Efficacy

As SASJ was first introduced in 2019, there are limited studies evaluating short-term efficacy and safety profile. A few published studies have shown that SASJ is a viable revisional procedure option for managing postoperative nutritional complications such as hypoalbuminemia and malnutrition that can arise after SASI [69,70]. Sewefy and Saleh reported an EWL of 85% at 1 year following SASJ. Within the first 3 months of SASJ, all T2DM patients achieved normalization of glucose levels with maintained resolution throughout the 2-year postoperative period, a trend similar to that in the SASI study conducted by Mahdy et al. [58]. The study found an 89% remission rate of hypertension and 100% improvement in all cases of dyslipidemia and OSA. A total of 87% of GERD patients reported improvement following SASJ. The study was the first on SASJ with 2 years of follow-up and concluded a 8.6% overall complication rate with complications of bile reflux/biliary gastritis (3.3%), dumping syndrome (2.7%), bleeding (1.3%), and leak (0.7%), and a 30-day readmission rate of 3.3% [71]. Regarding nutritional status, none of the patients developed protein malabsorption, anemia, hypoalbuminemia, or any other nutritional deficiency. These findings are consistent with the SASI study by Mahdy et al. [58,71].

## 8. Magnet System for Duodeno–Ileostomy (MSDI)

The magnet system for duodeno–ileostomy (MSDI) is a novel procedure used to treat weight loss, consisting of the formation of a minimally invasive magnetic compression duodeno–ileal anastomosis. This procedure is beneficial as a precise bowel separation, stitching, or staples is not required, and the magnets used in the procedure do not leave any trace in the patient’s body [14].

### 8.1. Technique

The MSDI procedure involves two main constituting parts: the magnetic rings and the magnet anastomosis delivery system (MADS). The magnetic rings are made of neodymium along with a titanium coating, making them biocompatible and durable. In addition to the magnetic rings’ strong material, suture loops made of stainless steel, nitinol, or polyester are attached to the rings, providing easier placement as well as recovery. The MADS is a flexible catheter for orogastric administration: it is made of a handle, a distal tip, and a positioning device assembly, which distribute and move the magnets in the right direction. This makes it possible for the magnets to be positioned at the correct location [72].

**Figure 6:**
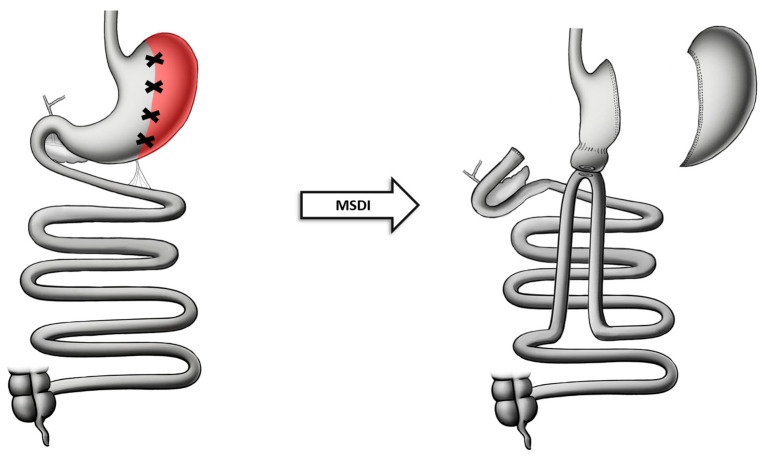
Anatomical configuration of MSDI.

Under general anesthesia, the surgeon places one marker in proximity to the cecum. Titanium clips and a bowel clamp distal to the ligament of Treitz are utilized to correct the position of the magnets. With the assistance of a flexible endoscope, the first (distal) magnet is placed in the proximal jejunum, which is attracted to the clamp (ligament of Treitz). The endoscope is withdrawn, and a position device is inserted to correct the magnet position more accurately. The position device removes the clamp and directs the magnet through the jejunum to the marked ileal position. The distal magnets are positioned anterior and latero–lateral to the duodenum. Once the distal magnets are accurately positioned, the proximal magnets are delivered endoscopically and aligned accordingly. The position device and the endoscope are withdrawn, and the mesenteric deviation is closed with silk sutures. This will slowly lead to the formation of an anastomosis through gentle ischemia within the tissue between the magnets over a 5- to 7-day period. In the 2–6 weeks following the formation of the anastomosis, the magnets slowly detach from each other and are naturally expelled from the patient’s body. Once the magnets are placed, an SG is performed with the help of an endostapler, followed by a leak test to ensure the strength and the integrity of the anastomosis [73] (Figure 6).

### 8.2. Safety and Efficacy

Gagner et al. examined the safety of this procedure on five patients with a BMI of ≥30 to ≤50 kg/m^2^ [73]: all magnets were successfully removed without any additional interventions, with an average expulsion time of 58.2 days. Additionally, all patients underwent an endoscopy demonstrating healthy mucosal tissue surrounding their patent duodeno–ileal anastomosis. There were a total of 16 adverse events: 10 Clavien-Dindo Classification (CDC) grade I adverse events (62.6%), 5 grade II events (31.2%), and 1 grade III event (6.2%).

Three of the five patients reported abdominal pain (CDC grade I) requiring intramuscular pain relief, one patient experienced minor upper esophageal mucosal rupture as a result of endoscopic overtube insertion (grade I), and another patient experienced an intra-abdominal hematoma next to the SG staple line (grade II). Both of these complications required no additional procedures or blood transfusions. A third patient experienced a serosal tear of the ileum as a result of the laparoscopic forceps pushing on the tissue during the operation (grade III); this was sutured and resolved the following day [73]. All patients required vitamin B12 supplementation at 180 days postoperatively due to vitamin B12 deficiency. Overall, the study reported no device-related adverse effects or any serious adverse effects or deaths [73].

In this preliminary study, mean TWL at 12 months was 34.0 ± 1.4%, EWL was 80.2 ± 6.6%, and BMI reduction was 15.1. Patients also experienced a drop in HbA_1c_ of 2.0 ± 1.6% at day 360 (*p* = 0.07), and a decrease in mean glucose levels from 134.3 mg/dL at baseline to 87.3 mg/dL (*p* = 0.07). A subsequent multicenter study with a cohort of 24 patients with BMI of ≥35 to ≤50 kg/m^2^ confirmed the promising outcomes, showing a mean TWL of 28.1 ± 1.0% and a mean EWL of 66.2 ± 3.4% at 6 months; and a TWL of 34.0 ± 1.4% and a mean EWL of 80.2 ± 6.6% at 12 months, with significant reductions in mean HbA_1c_ and glucose levels.

### 8.3. Conclusions

In conclusion, the side-to-side duodeno–ileal diversion with magnetic compression anastomosis is a novel technique with promising preliminary results, which shows potential for significant weight loss with minimal postoperative complications. Further studies are required to explore this concept further and evaluate outcomes in larger cohorts.

## 9. Conclusions

As we have seen, bariatric surgery has grown into a highly complex field of surgery, with novel procedures emerging and gaining popularity over historical ones. This reflects the growing incidence of obesity and the worldwide effort towards improving its treatment, together with the availability of newer technologies and a better understanding of the hormonal and molecular mechanisms underpinning obesity and obesity-related comorbidities.

## Figures and Tables

**Table 1:** Novel surgical techniques for the treatment of obesity.

Technique	Year of Introduction	Complications	Weight Loss	Comorbidity Resolution
SADI-S	2007	11.8% (early) 22.9% (late)	EWL 83.3% (1 year) TWL 37.5–38% (1 year) TWL 40.4% (5 years)	T2DM 75–89.5% Hypertension 68.3% Dyslipidemia 70.1%
OAGB	1997	8.1% (early) 12.4% (late)	EWL 89% (1 year) EBMIL 87.9% (2 years)	T2DM 76.9% Hypertension 64.3% Dyslipidemia 71.8%
SG-TB	2006	6% (early) 5.6% (late)	EBMIL 83.7–87.7% (1–2 years) TWL 20.2–54.7% (1–2 years)	T2DM 79.2–86% Hypertension 72–77% Dyslipidemia 85% OSA 88–91%
SASI	2016	12.3% (overall) 1.9% (major)	EWL 72.6–90% (1 year)	T2DM 83.9–97.9% Hypertension 36.1–70.4% Dyslipidemia 65% OSA 57.8–100%
SASJ	2019	8.6% (2 years)	EWL 85% (1 year)	Hypertension 89% Dyslipidemia 100% OSA 100%
MSDI	2023	6.2% (grade II CDC)	EWL 80.2% (1 year)	Hb_A1c_ drop 2.0 mg/dL

CDC: Clavien-Dindo Classification; EBMIL: Excess Body Mass Index Loss; EWL: Excess Weight Loss; MSDI: Magnet System for Duodeno-Ileostomy; OAGB: One-Anastomosis Gastric Bypass; OSA: Obstructive Sleep Apnea; SADI-S: Single-Anastomosis Duodeno-Ileostomy with Sleeve; SASI: Single-Anastomosis Sleeve-Ileal bypass; SASJ: Single-Anastomosis Sleeve-Jejunal bypass; SG-TB: Sleeve Gastrectomy with Transit Bipartition; T2DM: Type 2 Diabetes Mellitus; TWL: Total Weight Loss.

## Data Availability

Not applicable.
